# Peritoneal carcinomatosis secondary to metastatic lung cancer complicated with acute suppurative appendicitis: A case report and literature review

**DOI:** 10.1097/MD.0000000000031866

**Published:** 2022-12-09

**Authors:** Ji-Xin Fu, Xu-Jie Wang, Min Xia, Xin-Jian Wang

**Affiliations:** a Department of Gastrointestinal Surgery, Weihai Central Hospital, Weihai, Shandong, China; b Department of Ophthalmology, Weihai Central Hospital, Weihai, Shandong, China.

**Keywords:** acute suppurative appendicitis, case report, EGFR, lung cancer, peritoneal carcinomatosis

## Abstract

Lung cancer (LC) is a malignant tumor with the highest morbidity and mortality in the world. The most common metastatic sites of LC are the brain (47%), bone (36%), liver (22%), adrenal glands (15%), thoracic cavity (11%) and distant lymph nodes (10%). Peritoneal carcinomatosis (PC) is a rare clinical event in LC patients. Considering the rarity and nonspecific clinical symptoms of peritoneal metastasis among LC patients, a case of peritoneal metastasis secondary to LC incidentally observed by laparoscopic appendectomy is unusual.

**Patient concerns:**

Here, we present a 53-year-old never-smoker woman who presented to the emergency department with a 2-day history of pain in the right abdominal quadrant. Later, laparoscopy revealed acute suppurative appendicitis accompanied by a peritoneal metastatic mass.

**Diagnosis:**

The patient was diagnosed with PC secondary to metastatic LC complicated with acute suppurative appendicitis by immunohistochemistry. Positron emission tomography computed tomography (PET CT) findings further strengthen the evidence of PC from LC.

**Outcomes:**

Based on the results of genomic analysis, the patient received targeted therapy with osimertinib 80 mg/d.

**Lessons:**

Due to the discovery of new targets, the use of molecular therapies improved progression-free survival (PFS) and overall survival (OS), which increases the chance of identifying peritoneal metastasis of LC. For LC patients with abdominal symptoms, clinicians should be aware of the possibility of peritoneal metastasis from LC, especially for patients diagnosed with lung adenocarcinoma or with pleural effusion.

## 1. Introduction

Lung cancer (LC) is a malignant tumor with the highest morbidity and mortality in the world.^[1]^ Non-small cell lung cancer (NSCLC) accounts for 80% of all cases of LC, and approximately 75% of NSCLC cases are in an advanced stage at the time of initial diagnosis.^[2,3]^ Distant metastasis, as the main cause of treatment failure among LC patients, seriously affects the prognosis. Approximately 40% of LC cases are metastatic at diagnosis, with the most common sites of metastases being the brain (47%), bone (36%), liver (22%), adrenal glands (15%), thoracic cavity (11%) and distant lymph nodes (10%).^[4–6]^ Currently, diagnosis and treatment guidelines have been established for the common metastasis of NSCLC (such as brain, bone, liver and adrenal gland metastases). However, for uncommon metastases, treatment still faces great challenges. Peritoneal carcinomatosis (PC) is a rare clinical event in LC patients, with autopsy results showing an incidence of between 2.7% and 16%.^[7–9]^ In recent years, some large-scale studies have found that the incidence of peritoneal metastasis is lower than 5%.^[8,10,11]^ The prognosis of LC patients with peritoneal metastasis is very poor, and literature data have shown that the median overall survival (mOS) from the onset of PC with systemic therapies is less than 3 months.^[8]^

We report a case of peritoneal metastasis secondary to LC incidentally found by laparoscopic appendectomy and review the current literature to further characterize the clinical features, histology, and prognosis of PC secondary to LC.

## 2. Case report

**Figure 1.**
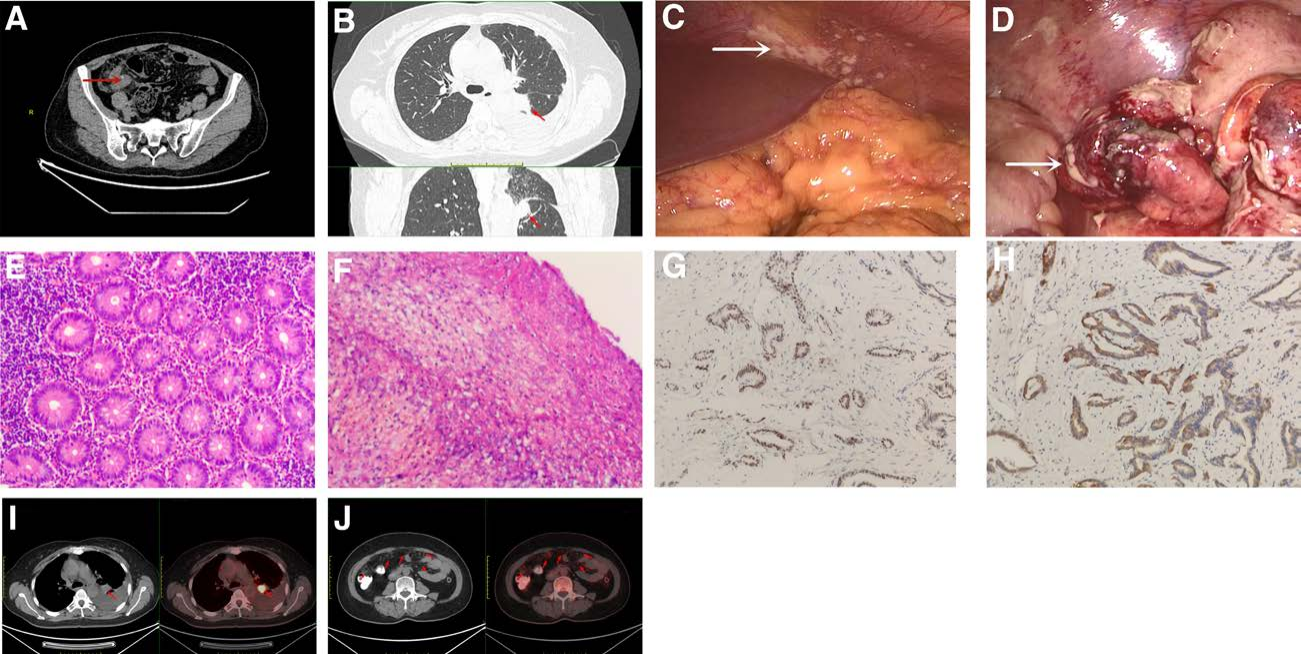
(A) Abdominal computed tomography (CT) demonstrated appendicitis with appendiceal calculi and surrounding inflammation secondary to appendicitis (red arrow). (B) Chest CT examination showed a nodule in the lower lobe of the left lung (red arrow). (C) Laparoscopic revealed a large number of diffuse miliary neoplastic implants on the peritoneum and omentum (white arrow). (D) Laparoscopic revealed an acute suppurative appendicitis accompanied by perforation (white arrow). (E) Pathology showed acute suppurative appendicitis with gangrene (H&E stain, 200 × magnification). (F) Pathology showed that infiltrating adenocarcinoma was found in the fibrous adipose tissue of the greater omentum (H&E stain, 200 × magnification). (G) Positive for TTF1 (TTF1 immunohistochemical stain, 200 × magnification). (H) Positive for CK7 (CK7 immunohistochemical stain, 200 × magnification). (I) PET/CT showed a hypermetabolic lesion in the lower lobe of the left lung (red arrow). (J) PET/CT showed hypermetabolic lesions in mesentery and omentum (red arrow).

A 53-year-old never-smoker woman presented to the emergency department with a 2-day history of pain in the right abdominal quadrant. She denied fever, vomiting, changes in bowel habits or urinary symptoms. She had a history of left salpingectomy for ectopic pregnancy 30 years ago. On physical examination, her temperature was 36.8°C, her heart rate was 71 bpm, and her blood pressure was 132/78 mm Hg. Her abdomen was soft. However, the right lower abdomen had obvious tenderness with rebound tenderness, which suggested clinical peritonitis. Laboratory work-up demonstrated a white cell count of 17.18 × 10^9^/L, neutrophil count of 89.9%, hemoglobin level of 151 grams per liter, and platelet count of 287 × 10^9^/L. Abdominal computed tomography (CT) demonstrated appendicitis with appendiceal calculi and surrounding inflammation secondary to appendicitis (Fig. 1A). Chest CT examination showed a nodule in the lower lobe of the left lung, with a diameter of approximately 10 mm and unclear boundary. There was a small amount of pleural effusion in the left lung (Fig. 1B).

Considering acute appendicitis with peritonitis, an emergency laparoscopic operation was performed. At laparoscopic operation, there was approximately 500 mL purulent ascites in the abdominal cavity, and a large number of diffuse miliary neoplastic implants were found on the peritoneum and omentum (Fig. 1C). The appendix, which was gangrene accompanied by perforation, was wrapped by the greater omentum and small bowel to form an abscess (Fig. 1D). Due to the possibility of peritoneal metastasis, laparoscopic appendectomy, omental biopsy and abscess debridement were performed. Pathology showed acute suppurative appendicitis with gangrene (Fig. 1E), and infiltrating adenocarcinoma was found in the fibrous adipose tissue of the greater omentum, which confirmed the diagnosis of metastasis from adenocarcinoma (Fig. 1F). Immunohistochemical analysis: MLH1 (+), PMS2 (weak +), MSH2 (+), MSH6 (+), HER2 (0), CK7 (+), CK20 (−), cdx20 (−), Pax8 (−), WT-1 (−), TTF-1 (+), Cr (−) (Fig. 1G and H). Postoperative positron emission tomography computed tomography (PET CT) further strengthened the evidence of PC from LC (Fig. 1I and J). Genomic analysis was performed to identify whether there were any driver mutations present, and it revealed an epidermal growth factor receptor (EGFR) mutation in exon 21 L858. The patient was given osimertinib 80 mg/d, and after 3 months of follow-up, she was disease free.

The study was reviewed and approved by Ethical Committee of Weihai Central Hospital. Written informed consent was obtained from the patient for publication of this case report and any accompanying images.

## 3. Discussion

The most common malignancies that spread along the peritoneum are from the gastrointestinal tract and ovary. PC from LC is extremely rare,^[8,10,11]^ especially in those at the time of initial diagnosis. Although autopsy results showed an incidence of between 2.7% and 16% in the early years,^[7–9]^ the clinical incidence was much lower. Diagnosis of PC is clinically difficult unless the patient is terminally ill or is already known to have massive ascites. Satoh et al^[12]^ reviewed 1041 LC patients over a 26-year period, of whom 1.2% developed clinically apparent PC. We report an unusual peritoneal metastasis of LC that was occasionally found during laparoscopic appendectomy for acute appendicitis, which was rarely reported in the previous literature.

Despite chemotherapy, the outcome of LCs with PC is very poor, and the median survival is only 12 weeks.^[8,13]^ Considering the lack of management guidelines for peritoneal metastasis from LC, we searched the PubMed and WanFang databases using the terms “lung cancer” with “peritoneal carcinomatosis” or “peritoneal Metastases” or “ascites” and reviewed the current literature to further characterize the clinical features, histology, and prognosis of PC secondary to LC, expecting to provide experience for future treatment. We identified 8 retrospective studies and 5 case reports involving as many as 311 patients. To the best of our knowledge, this is the largest review of the literature about peritoneal metastasis of LC, and the basic characteristics of these patients are shown in Tables 1 and 2.

**Table 1:** Basic characteristics of peritoneal carcinomatosis secondary to metastatic lung cancer in review of literature.

Author	Date	n	Average age	Gender	Smoking status	Pleural effusion	Histology	mOSl (mo)	mOS2 (mo)	Country
Shinozaki T^[14]^	2018	1	76	M/1	0/1	1/1	AD/1	0.75	0.25	Japan
Hsu JF^[15]^	2015–2017	3	62 (range, 53–67)	male to female 2:1	1/3	3/3	AD/3	23.67	7.67	China（taiwan）
Nassereddine H^[13]^	2007–2016	12	58 (range, 45–83)	male to female 9:3	12/12	No record	AD/11; SCC/1	12.75	3	France
Kobayashi H^[16]^	2016	1	61	F/1	0/1	1/1	AD/1	108	12	Australia
Patil T^[11]^	2008–2015	33	58 (range, 41–91)	male to female 12:21	20/33	No record	NSCLC/33	18.5	2	United States
Hanane K^[17]^	2016	1	56	F/1	1/1	No record	AD/1	16	2	Morocco
Satoh H^[12]^	1976–2001	12	54 (range, 34–74)	male to female 6:6	No record	9/12	AD/7; SCC/2; Large/2; SCLC/1	11	2	Japan
Su HT^[8]^	1990–2005	30	59 (range, 29–83)	male to female 20:10	No record	24/30	AD/25; SCC/1; SCLC/3; mixed AD and SCC 1	9	0.5	China（taiwan）
Abbate MI^[18]^	2014–2015	60	60 (range, 25–75)	male to female 44:16	43/60	No record	AD/48; SCC/1; other/11	17.5	3.53	Italy
Sibio S^[19]^	2019	2	52.5 (44 and 59)	M/2	1/2	0/2	AD/2	72.5	30.5	Italy
Zhao BJ^[20]^	2014–2019	5	47.5 (36–75)	male to female 2:3	No record	4/5	AD/5	14.4	8.3	China
Cao B^[7]^	2010–2018	12	47.5 (36–75)	male to female 8:4	No record	6/12	AD/12	18	3	China
Flanagan M^[21]^	1994–2012	139	-<50 8 -50–59 29 -60–69 51 -70–79 78 -80 + 31	male to female 80:59	No record	No record	AD/51; SCC/21; SCLC/27	2.3	1.3	Ireland

No record means no description in the published article.

AD = adenocarcinoma, mOS1 = median overall survival from non-small-cell lung cancer diagnosis until death for any cause or last follow-up, mOS2 = median overall survival from diagnosis of peritoneal carcinomatosis until death for any causes or last follow-up, NSCLC = non–small cell lung carcinoma, PC = peritoneal carcinomatosis, SCC = squamous cell carcinoma, SCLC = small cell lung cancer.

**Table 2:** Gene mutation characteristics of peritoneal carcinomatosis secondary to metastatic lung cancer in review of literature.

Author	n	EGFR	ALK	TP53	KRAS	STK11	BRAF	DDR2	ERBB4	SMAD4	CTNNB1	ROS1	MET
Shinozaki T^[14]^	1	0/1	0/1	/	/	/	/	/	/	/	/	/	/
Hsu JF^[15]^	3												
	No.1	1/1	/	/	/	/	/	/	/	/	/	/	/
	No.2	1/1	/	/	/	/	/	/	/	/	/	/	/
	No.3	1/1	/	/	/	/	/	/	/	/	/	/	/
Nassereddine H^[13]^	12	1/9	/	5/9	3/9	2/9	2/9	1/9	1/9	1/9	1/9	/	/
Kobayashi H^[16]^	1	1/1	/	/	/	/	/	/	/	/	/	/	/
Patil T^[11]^	33	17/33	5/33	/	5/33	/	0/33	/	/	/	/	/	1/33
Hanane K^[17]^	1	0/1	/	/	/	/	/	/	/	/	/	/	/
Abbate MI^[18]^	60	7/23	3/17	/	/	/	/	/	/	/	/	1/3	2/4
Zhao BJ^[20]^	5	5/5	/	/	/	/	/	/	/	/	/	/	/
Cao B^[7]^	12	7/12	/	/	/	/	/	/	/	/	/	2/12	/

“/” means no description in the published article.

The whole literature review spans 1976 to 2019, involving 311 patients with PC secondary to LC. The data came from 9 countries or regions, such as China, Japan and France. The age of the patients ranged from 29 to 91 years old, mainly middle-aged and elderly people. Males comprised over half of the patients with LC who were diagnosed with PC (n = 186/311; 59.8%). Sixty-nine percent (78/113) of patients who developed peritoneal metastasis were current or former smokers, which was consistent with the results of JNassereddine H.^[13]^ Patil T et al^[11]^ found that the majority of patients who smoked had either KRAS or no identifiable mutations, and patients with ALK and EGFR mutations tended to be never-smokers.

In our literature review, the most common histological type of LC associated with PC was adenocarcinoma, accounting for 60.01% (167/278), and as many as 70% (48/68) of the patients with data records were complicated with pleural effusion, suggesting that lung adenocarcinoma and pleural effusion may be risk factors for peritoneal metastasis of LC. Possible reasons are as follows: pulmonary adenocarcinoma is prone to hematogenous metastasis, and hematogenous metastasis is one of the main mechanisms of PC in NSCLC; pulmonary adenocarcinoma has a high proportion of gene mutations, and targeted therapy not only improves the prognosis of patients but also prolongs the time for tumor cells to adapt to the peritoneal microenvironment, increasing the probability of PC; the human diaphragm has naturally extensive lymphatic networks consisting of stomata, lymphatic vessels, and lacunae.^[22–25]^ Takagi et al^[26]^ found that the diaphragm has rich lymphatic networks in patients with lymphangioleiomyomatosis and that chyloid effusion can penetrate into the abdominal cavity through the lymphatic vessels of the diaphragm lesions. Therefore, among patients with malignant pleural effusion, it is possible that the tumor will invade the diaphragm, which can lead to PC through lymphatic metastasis.

Several genetic mutations appear to be associated with an elevated risk of LC. However, the relationship between gene mutation and peritoneal metastasis of LC is still unclear. In our retrospective study, EGFR was the most frequently mutated gene, accounting for 48.24% (41/85). Other genes with more mutations were KRAS and ALK, accounting for 19.059 (8/42)% and 35% (8/51), respectively. Patil et al^[11]^ found ALK rearrangement and EGFR, KRAS, and MET mutations in 15%, 52%, 15%, and 3% of patients, respectively, which was nearly consistent with our results. Mutations in EGFR are common oncogenes in LC, such as the T190 M mutation in EGFR, which was found to be the most prevalent in NSCLC patients with drug resistance to tyrosine kinase inhibitors (TKIs), such as gefitinib and erlotinib.^[27]^ KRAS, the most common driver mutation in patients with NSCLC, was the second most frequently mutated gene. Mutations in the KRAS gene are not yet targetable and confer a poor prognosis in the metastatic setting.^[28,29]^ In our retrospective study, TP53 was one of the most frequently mutated genes. As reported in the literature, these mutations were associated with poorer survival and poorer response to radiation and adjuvant cisplatin therapies.^[30]^ The frequency of TP53 gene mutation was lower than those of other mutations in some of our cases. This may be a representation of tumor heterogeneity and the selection and adaptation occurring during metastatic disease progression.^[31]^

Because no systematic body of knowledge on the diagnosis and treatment of PC secondary to primitive lung carcinoma is available, the treatment strategies for the patients were different in our retrospective study, leading to varied overall survival (OS). Some reports have shown survival benefits for select patients receiving aggressive chemotherapy^[9]^; however, most patients cannot tolerate aggressive chemotherapy because of poor performance conditions and short life expectancy. Satoh et al^[12]^ reported a very poor prognosis if the patients did not receive aggressive chemotherapy. Su et al^[8]^ also showed a very poor prognosis (median 15 days). In their subgroup analysis, there was a significant difference in survival between the groups that received conservative or aggressive management (14.7 days vs 127.3 days, *P* < .001). Two patients who responded to gefitinib therapy had improved abdominal conditions and a gradual decrease in ascites, and they survived for 203 and 343 days, respectively, in marked contrast to the poor median survival of 15 days. Sereno et al^[9]^ documented 4 case reports in which the patients received chemotherapy with pemetrexed + cisplatin, pemetrexed + carboplatin, pemetrexed and docetaxel. Among these cases, the patient who received pemetrexed and cisplatin had a relatively long progression-free time of 5 months. Beyond chemotherapy, some patients have been reported to have a good response to EGFR-TKIs, with improvements in abdominal condition and a gradual decrease in ascites with longer survival. Kobayashi et al^[16]^ reported a case of lung adenocarcinoma with recurrent malignant ascites after 10 lines of therapy. The patient finally received afatinib and had a progression-free survival (PFS) of more than 12 months. The researchers also reported another patient who had a good response to erlotinib. Hsu JF et al^[15]^ showed that 3 patients all responded to bevacizumab-based therapy, and 2 had at least 5 months of PFS. However, the role of antiangiogenic agents in the management of malignant ascites is still controversial.

Most patients with LC have peritoneal metastasis in the final clinical event stage, which is often not an isolated peritoneal metastasis but a multiple systemic metastases. In our patient, PET CT showed that the LC had been accompanied by rib and lymph node metastasis at the same time as peritoneal metastasis. Despite chemotherapy or targeted therapy, the outcome of LCs with PC was very poor, as reported in our retrospective study (mOS2 in Table 1). It is reported that the mOS from the onset of PC with systemic therapies is less than 3 months.^[8]^ Our patient received targeted therapy with osimertinib, and after 3 months of follow-up, she was disease free.

There are several limitations to this study. First, the number of included cases was small. Regardless, this is the largest literature review of PC derived from LC to date. Second, the treatment results were heterogeneous even though a comprehensive literature review was performed. The quality of the evidence was low since all of the studies were case reports or case series studies, in which many clinical data were not recorded. Therefore, the clinical treatment strategies were inconclusive. Third, the study spanned a long period of time. Antitumor drugs and treatment strategies have been updated many times in this period, which made the treatment efficacy hard to evaluate.

## 4. Conclusion

In conclusion, we reported a case of peritoneal metastasis secondary to LC accidentally found by laparoscopic appendectomy, and a comprehensive literature review was performed to summarize the clinical features, histology, and prognosis of PC secondary to LC. Moreover, further large-scale clinical trials are warranted to provide useful information to clinicians on how to treat PC derived from LC.

## Author contributions

**Data curation:** Ji-Xin Fu.

**Methodology:** Min Xia.

**Project administration:** Xu-Jie Wang.

**Writing – original draft:** Ji-Xin Fu.

**Writing – review & editing:** Xin-Jian Wang.
